# *Nigella sativa* L. as immunomodulator and preventive effect on renal tissue damage of lupus mice induced by pristane

**DOI:** 10.1016/j.heliyon.2022.e09242

**Published:** 2022-04-06

**Authors:** Zahrah Hikmah, Anang Endaryanto, I. Dewa Gede Ugrasena, Anny Setijo Rahaju, Syaiful Arifin

**Affiliations:** a Medical Doctoral Program Student, Faculty of Medicine, Universitas Airlangga, 60132, Surabaya, Indonesia; b Division of Allergy and Immunology, Departments of Pediatric of Medicine Universitas Airlangga, Surabaya, Indonesia; c Dr. Soetomo Academic Teaching Hospital, 60286, Surabaya, Indonesia; d Universitas Airlangga Academic Hospital, Surabaya, Indonesia; e Department of Anatomical Pathology, Faculty of Medicine Universitas Airlangga, Surabaya, Indonesia; f Postgraduate School of Biomedicine, Faculty of Medicine, Universitas Brawijaya, Malang, Indonesia

**Keywords:** *Nigella sativa* L., Immunomodulator, Lupus, Renal

## Abstract

**Introduction:**

*Nigella sativa* L. is an herbal plant with Thimoquinone as the main therapeutic properties. This plants has been shown to cure for various diseases and affected the immune system by modulating cytokines and T regulatory cell (Treg) sot that able to prevent renal injury in several diseases, but studies on Systemic Lupus Erythematous are still rare.

**Objective:**

This study aimed to investigate immunomodulation and preventive effects of *Nigella sativa* L. on renal tissue damage in Pristane induced Lupus (PIL)-mice model**.**

**Methods:**

This true experimental study included 48 female Balb/C mice, 38 mice were injected pristane intraperitoneally and waited 16 weeks to become lupus model. Only 30 mice met the Systemic Lupus International Collaborating Clinics criteria. Ten healthy mice were used as control, 30 PIL mice model divided into 3 groups (placebo, steroid, *Nigella sativa* L.). At the end of 28 days of treatment, the mice were sacrificed to take a blood sample and kidney organ to evaluate the injury histopathologically.

**Results:**

The results showed that the cytokine expression Interleukin (IL) (IL-17, IL-6, IL-23) in the *Nigella sativa* L. group was the lowest. The highest absolute number of Tregs was the steroid group followed *Nigella sativa* L. group. Renal injury assessed histopathologically showed the *Nigella sativa* L. group was the lowest and almost close to normal.

**Conclusion:**

This study indicate that *Nigella sativa* L. has an immunomodulatory effect and can prevent kidney injury PIL-treated mice. We suggest that *Nigella sativa* L. may need to be considered for further research on its use as a complementary supplement in lupus patients.

## 1 Introduction

Systemic Lupus Erythematous (SLE), is a chronic autoimmune disease, with the main problem is the dysregulation of the immune system and its response to self-antigens, causing damage to various organs and tissues (La Paglia et al., 2017; Kaul et al., 2016). In some studies, the prevalence of renal injury in SLE is 41–55% (Hiraki et al., 2008; Osio-Salido and Manapat-Reyes, 2010; Hamijoyo et al., 2019), is mainly associated with the disorder of the immune system, deposition of immune complexes in the tissues resulting in inflammation and secretion of pro-inflammatory cytokines (Yap and Lai, 2010; Cunha and Gilek-Seibert, 2016). Consequently, alternative treatment is needed to protect the renal, such as an immunomodulator. Immunomodulation is interpreted as a temporary alert in certain parts of the immune system which can change caused by the agents that activate or suppress its function, and acts to regulates the immune system (Wen et al., 2012).

Glucocorticoid and immunosuppressant are still the main drugs that signify applied in SLE with broadside effects that are not specific, so that long-term use will cause severe problems for SLE patients and even lead to death (Kudo, 2015; Giraldo et al., 2012; Amissah-Arthur and Gordon, 2010). Lupus drug side effects vary widely depending on dosage and the length of treatment. For a long time using, it leads to clinical complications such as secondary infection, cataracts, musculoskeletal diseases including osteoporosis, vascular necrosis, and myopathy, and also cardiovascular complications such as myocardial infarction and cerebrovascular disease (Oon et al., 2018; Sinha and Bagga, 2008; Kasturi and Sammaritano, 2016). It is necessary to find an alternative source with effective, safe, cheaper immunomodulator agents for SLE patients.

*Nigella sativa* L., also known in Indonesian as Habatussauda or Jinten Hitam (Black Cumin), has been utilized for resolving numerous diseases since hundreds of years ago and was generally used in traditional medicine (Kabir et al., 2020)*. Nigella sativa* L., seeds contain fixed oil, proteins, alkaloids, saponins, and essential oil. There are variant of compounds present in *Nigella sativa* L., including thymoquinone (TQ), thymohydroquinone, dithymoquinone, p-cymene, α-thujene, γ-terpinene, carvacrol, α-pinene, β-pinene, 4-terpineol, and sesquiterpene longifolene, carvone, limonene, and citronellol. TQ is one of the active compounds in *Nigella sativa* L. and it has been widely investigated in various diseases. Previous studies of *Nigella sativa* L. showed antimicrobial, antibacterial, antifungal, antiparasitic, anticancer, anti-Inflammatory, and immunomodulatory activities (Kabir et al., 2020; Islam et al., 2017). The biological effects of *Nigella sativa* L. are attributed to the various experimental studies on animal models have proven that it acts as the immunomodulator in autoimmune diseases by reducing cluster of differentiation (CD)8+ and increasing the percentage of CD4+ CD25+ T cells in rheumatoid arthritis (RA) patients (Kheirouri et al., 2016). It also proved to increase the percentage of Treg and decreased T helper (Th)17 in asthma mice models (Barlianto et al., 2012). *Nigella sativa* L. powder can modulate the immune response by lowering IL-23 in people with Hashimoto's thyroiditis (Tajmiri et al., 2016) and production of autoantibodies in several other autoimmune diseases (Hmza et al., 2013; Farhangi et al., 2016). Moreover, it can also be used to protect the kidney from various diseases. However, the immunomodulatory and preventive effect of *Nigella sativa* L. in renal tissue of SLE patient has not been studied in a systematic context. Therefore, this study was done to evaluate the immunomodulator and renal tissue preventive effect of *Nigella sativa* L. extract in the lupus mice model.

## 2 Material and methods

### 2.1 Animals

BALB/c female mice (age 6–8 weeks, 20–30 g) have been supplied from Universitas Islam Negeri Malang, Indonesia. Animals were adapted for one week before starting the experiments under appropriate temperature, humidity, and light conditions. They were fed a common diet and very free access to libitum ad air. All mice were maintained at the Pharmacy Laboratory, Faculty of Medicine, Universitas Brawijaya, Indonesia.

### 2.2 Materials

*Nigella sativa* L. was obtained from Materia Medica Batu, Malang, Indonesia, and extracted at the Pharmacy Laboratory, Universitas Airlangga, Indonesia. *Nigella sativa* L. seeds were ground and then extracted with ethanol solvent using the soxhletation method. The resulting mixture was vortexed for 1 min and sonicated for 20 min. In addition, incubated with ethanol solution for 24 h in a constant rotamix and Soxhlet machine, then vortexed again for 1 min and centrifuged for 25 min at 1400 rpm. The content of TQ in the extracted *Nigella sativa* L. was examined for levels using the High-Performance Liquid Chromatography (HPLC) method. The centrifuged supernatant of 20 L was injected into the HPLC machine in the water-ethanol mobile phase (25 + 75, v/v) with a flow rate of 1 mL/min. Quantification was achieved by UV-Vis Spectroscopy detection at 254 nm. A 0.2%. Sodium Carboxymethyl Cellulose (Na CMC) solution was prepared by weighing 40 mg of Na CMC. Prepare about 20 ml of hot water in a mortar. Sprinkle 40 mg of Na CMC into 20 ml of hot water in a mortar and grind it well. This liquid is used to disperse steroids according to the dose used then given with a forced feeding as much as 0.5 ml. Pristane was given 0.5cc intraperitoneally for each mouse and was obtained from Sigma-Aldrich, Singapore for this study.

### 2.3 Experimental design

The study design was a randomized post-test-only control group design. The PIL-treated mice are developed within 16 weeks after a 0.5 cc single intraperitoneal pristane injection and fulfill Systemic Lupus International Collaborating Clinics (SLICC) criteria (alopecia, arthritis characterized by swelling of 2 or more joints, and increased anti-dsDNA autoantibodies). Pristane can induce systemic lupus with a variety of symptoms such as organ involvement and autoantibodies in a variety of mouse strains. Pristane intraperitoneal injection will stimulate the formation of autoantibodies associated with lupus in the face of multiple nuclear antigens (Yan et al., 2020; Freitas et al., 2017; Li et al., 2017). BALB/c female mice were divided into four groups which are categorized into healthy group and 3 treatment groups. The treatment groups are Placebo group (PIL+ Na CMC 0.2% for 28 days-intragastrical), Steroid group (PIL + Prednisone 0.346 mg-0,369 mg (converted from human dose 1 mg/kg + Na CMC 0.2% for 28 days-intragastrical) and *Nigella sativa* L. group (PIL+ extract *Nigella sativa* L. 4.8 g/kg/day + Na CMC 0.2% for 28 days-intragastrical). On the first day of the 21st-week treatment, the mice were sacrificed to take the blood sample and to remove the renal organ for blood and histopathological evaluation.

### 2.4 Blood evaluation

The autoantibody concentrations against double stranded deoxyribonucleic acid (dsDNA) were measured by enzyme-linked immunosorbent assay (ELISA) microplate reader. Flat-bottomed 96-well plates coated with recombinant human dsDNA, sample buffer, wash buffer, tetramethylbenzidine substrate, and stop solution (1M HCl) were provided by Aesku Diagnostics (Wendelsheim, Germany). An antibody detection (horseradish peroxidase-conjugated goat anti-mouse IgG) was purchased from Chemi-Con. Diluted probes (1:500 and 1:5000; standard probes were continuously diluted in sample buffer to set a standard curve) were incubated for 1 h at room temperature and, after 3 washing steps, were subsequently incubated with detection antibody (peroxidase-labeled goat anti-mouse Immunoglobulin (Ig)G, dilution 1:1000 in diluent buffer, Chemicon) for 15 min at room temperature. After 3 additional washing steps, 100 μl of tetramethylbenzidine substrate was added. After 15 min, the reaction was stopped by adding 100 μl of stop solution (1M HCl). Finally, optical density was determined with an ELISA reader (Rainbow reader; SLT Labinstruments, Groding, Austria) at the wavelength of 450 nm. Analyses were performed in duplicate treatment. Anti-dsDNA titers are given as units per milliliter. IL-6 expression was measured based on the count relative percentage of IL-6 expressed by macrophage using IL-6 PE/Cy5.5-conjugated anti-mouse IL-6 (trademark Clone NBPI, Novus biologicals, LLC). CD11b is the marker of macrophage surface cells, were measured using CD11b FITC anti-mouse/human CD11b (clone: M1/70, BioLegend). The measurement of IL-6 was conducted using flow cytometry. IL-17 expression was measured based on the count relative percentage of IL-17 expressed by Th-17 using IL-17 marker PerCP/Cy5.5 anti-mouse IL-17A (Trademark Clone TC11-18H10.1, BioLegend). CD4 is the marker of T helper (Th) cells surface, were measured using FITC anti-mouse CD4 (Trademark Clone H129.19, BioLegend). The measurement of IL-17 was conducted using flow cytometry. IL-23 expression was measured based on the count relative percentage of IL-23 expressed by macrophage using PE-conjugated rat antimouse IL-23 (Trademark Alexa Fluor 488, eBioscience). The measurement of IL-23 was conducted using flow cytometry. CD11b is the marker of macrophage surface cells, were measured using CD11b Monoclonal Antibody (trademark Thermo Fisher Scientific, BioLegend). The Treg cells were count based on the percentage of absolute count of Treg cells: CD4 + CD25 + FoxP3 + IL-10 multiply by total lymphocyte cells. CD4 were measured using FITC anti-mouse CD4 (clone H129.19, BioLegend), CD25 were measured using PE anti-mouse CD25 (clone: 3C7, BioLegend), FoxP3 were measured using Alexa FluorR 647 anti-mouse/rat/human FOXP3 (Trademark Clone: 150D, BioLegend), and IL-10 were measured using PE/Cy7 anti-mouse IL-10 (trademark Clone: JES5-16E3, BioLegend).

### 2.5 Histopathology findings

Renal of mice were fixed by immersing them in 10% Neutral buffered formalin (NBF) solution for 24 h and cutting the tissue with a maximum size of 1 × 1 cm. Tissue was taken from 10% NBF solution and placed to the 70%, 80%, 90% alcohol solution for 1 day, respectively. Then, it is purified and replaced with xylol chemicals that possibly mixed with dehydrants and paraffins. The tissue was immersed in the xylol solution 2 times for 30 min (xylol I 30 min, xylol II 30 min). Then do immersion (infiltration/impregnation/embedding). The tissue was put in liquid paraffin at 56–60 °C for 3 × 1 h in an incubator (paraffin I 1 h, paraffin II 1 h, paraffin III 1 h). After Blocking/Casting, the tissue was inserted into the printer and then put into the cube and paraffin chamber. The paraffin blocks were stored overnight and sliced using a microtome with a thickness of 5–10 m and then placed in a water bath at 50 °C. After the paraffin tape was well developed, attach the paraffin tape to an object-glass coated with albumin + glycerin, dry and Hematoxylin and Eosin (HE) and Periodic acid–Schiff (PAS) staining processes. The preparations were affixed with a cover glass which had been added with a single drop of Canadian balsam. The preparations were viewed using a binocular microscope Then, light microscopy observations make under a binocular microscope—the finding record at the initial magnification of x400. The degree of renal damage was based on histopathology, while the classification was based on the International Society of Nephrology and the Renal Pathology Society (ISN/RPS) classification of Lupus Nephritis (LN) (Pinheiro et al., 2019; Sada and Makino, 2009). Organ fragments are printed by a pathologist expert using the LN activity index (AI) method by the National Institutes of Health (NIH). NIH-AI is a semi-quantitative grading system of pathologic features on renal biopsies that allows for monitoring response for treatment and showing disease progression. LN disease activity can be assessed on a renal biopsy using the modified NIH activity and chronicity indices. Indicators of disease activity involve endocapillary hypercellularity, neutrophils or karyorrhexis within glomerular capillary loops, fibrinoid necrosis, hyaline deposits, cellular or fibro cellular crescents, and interstitial inflammation. Crescents and fibrinoid necrosis are weighted twice as they have a worse impact on prognosis. The scoring is referred to the percentage of glomeruli with each feature in the biopsy on a scale of 0–3, with a score of 0 = not present, 1 = <25% glomeruli, 2 = 25–50% glomeruli, and 3 indicating >50% glomeruli. NIH-AI was used to measure the progression of LN (Bajema et al., 2018; Fava and Petri, 2019).

### 2.6 Assessment of CD34 cells by flow cytometry

All spleen is crushed and homogenized in a mortar using the sterile pestle as well as a fine mesh metal sieve inside a Petri dish added with phosphate buffer salt (PBS). Then, the suspension of the existing cells is inserted into the Falcon tube and centrifuged (2500 rpm, for 5 min at 10 °C) with a fried centrifuge (Hermle Z326K). Then eliminate the supernatant. Pellets containing spleen are inserted into microtubes with PBS and dicryopreserved using fetal bovine serum (FBS) and dimethyl sulfoxide (DMSO) with a maximum duration of 14 days until further evaluation using flow cytometry. Showing the flow cytometry experiments, so splenocytes unfroze. Resuspend using RPMI medium 1640 at room temperature, then rinsed and centrifuged (1500 rpm for 10 min at 4 °C) twice, then resuspended using FBS and PBS. Stained samples are stored for 1 h in darkrooms at 4 °C–8 °C with CD34 anti-mouse monoclonal Abs (BD Biosciences). Cells were permeabilized using Biolegend as permeabilization buffer with a ratio of 1:10 for dilution on stains for intracellular Foxp3. Shortly after incubation with monoclonal abs, then the sample is centrifuged and washed using PBS and then repaired using 4% paraformaldehyde. Then conducted flow cytometry analysis using flow cytometer BD FACS Calibur.

### 2.7 Statistical analysis

Statistical analysis was conducted using SPSS version 25. Normality test and homogeneity test was done to determine the data distribution using Shapiro-Wilk test, and continue with: The one-way ANOVA, for a normal variable with distribution and homogenous, post hoc using LSD multiple comparison Kruskal-Wallis test, for normal distribution but not homogenous, post hoc analysis using the Mann-Whitney U test.

### 2.8 Research ethics

This study was performed under ethical standards for animal experimentation, and the Ethical Committee approved it, Faculty of Veterinary Medicine, Universitas Airlangga, Indonesia (Ethical Clearance No. 2.KE.165.08.2019).

## 3 Results

Forty-eight mice were recruited and 38 mice were injected with pristine-intraperitoneal. Only 30 mice still alive and have been successfully fulfilled SLICC criteria lupus. Ten mice were used as healthy group. The profile of animal subjects was summarized as follows.

### 3.1 Serum levels of anti-dsDNA antibodies

**Figure 1:**
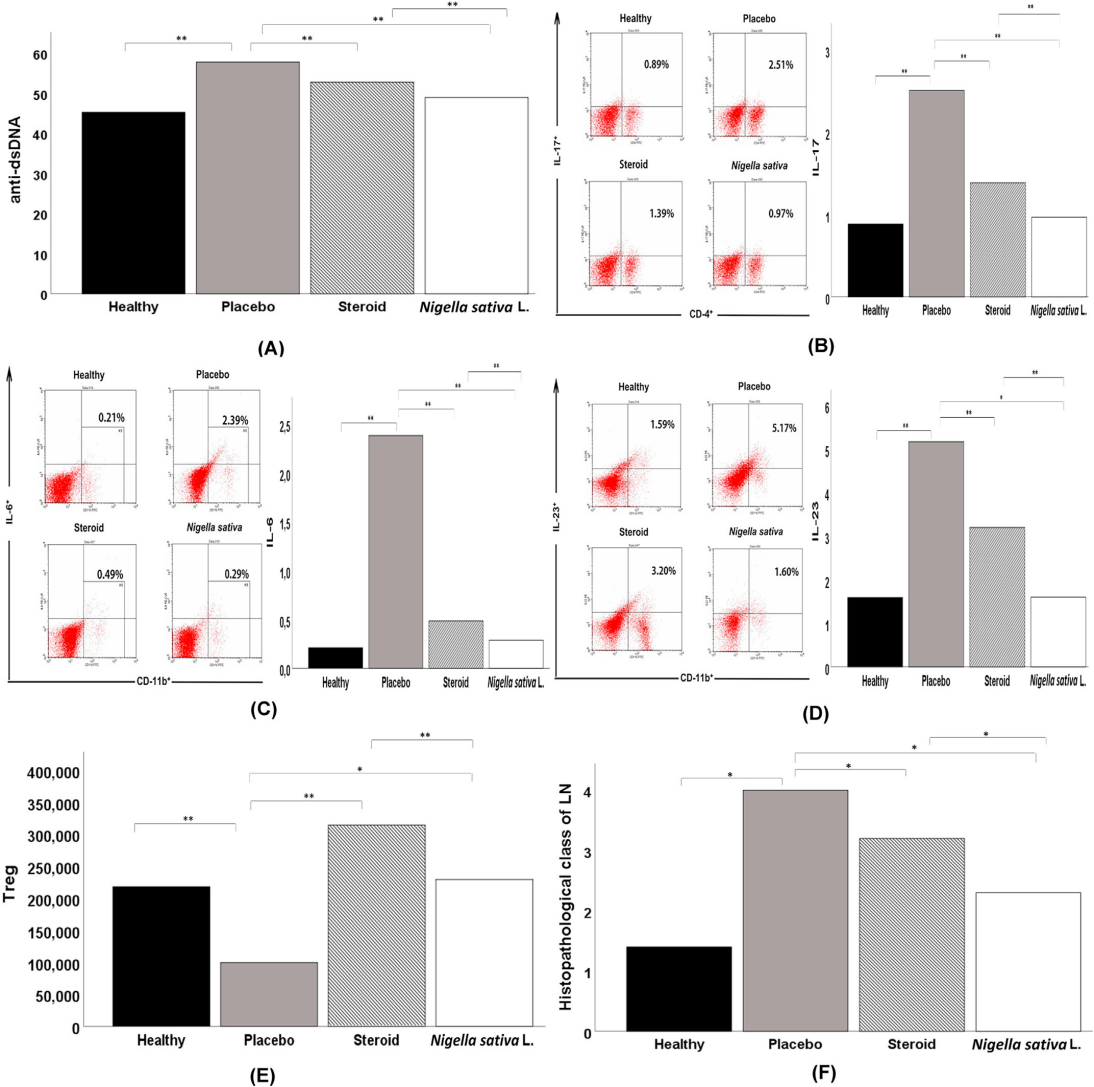
A) Anti-dsDNA analysis of all experimental groups, B) IL-17 analysis of all experimental groups, C) IL-6 analysis of all experimental groups, D) IL-23 analysis of all experimental groups, E) Treg analysis of all experimental groups, F) Histopathological Class of LN analysis of all experimental groups. Significant effect is indicated by asterisk (∗, p < 0.05; ∗∗, p < 0.001).

The mean results of measuring serum levels of anti-dsDNA antibodies in Healthy, Placebo, Steroid, and *Nigella sativa* L. groups are presented in Table 2 and Figure 1. The mean serum levels of anti-dsDNA antibodies were higher in the Placebo group compare to the Healthy, Steroid, and *Nigella sativa* L. groups. From the results o, a p-value of <0.001 showed that there were at least two groups that were significantly different. From the results of the test between groups, the mean levels were significantly higher in the Placebo group (57.88 ± 0.84) compared to the Healthy group (45.32 ± 2.56; p < 0.001), Steroids (52.86 ± 1.80; p < 0.001) and *Nigella sativa* L. group (49.01 ± 1.17; p < 0.001). Anti-dsDNA antibody levels in the *Nigella sativa* L. group were significantly lower compared to the Steroid group (p < 0.001).

### 3.2 IL-17 expression of lupus mice models

The IL-17 expression represents as the percentage of CD-4^+^ IL-17^+^ as summarized at Figure 1 and Table 2. The percentage of IL-17 expression is significantly higher in Placebo group (2.51 ± 0.72) compared to Healthy (0.89 ± 0.52, p < 0.001), Steroid (1.39 ± 0.27, p < 0.001) and *Nigella sativa* L. group (0.97 ± 0.31; p < 0.001). The percentage of IL-17 expression is lower at *Nigella sativa* L. group than Steroid group (0.97 ± 0.31 vs 1.39 ± 0.27 vs., p < 0.05).

### 3.3 The IL-6 expression of lupus mice models

The result of the IL-6 expression is presented as the average percentage of CD-11b^+^ IL-6^+^, as summarized in Table 2 and Figure 1. The placebo group has a higher IL-6 level compared to the Healthy, Steroid, and *Nigella sativa* L. group (p < 0.001). Expression of IL-6 was higher at Placebo group (2.39 ± 0.50) compared to Healthy (0.21 ± 0.09) Steroids (0.49 ± 0.07; p < 0.001) and *Nigella sativa* L. group (0.29 ± 0.06, p < 0.001). IL-6 expression on the *Nigella sativa* L. group was notably lower than the Steroid group (0.29 ± 0.06 vs 0.49 ± 0.07, p < 0.001).

### 3.4 The IL-23 expression of lupus mice models

**Figure 2:**
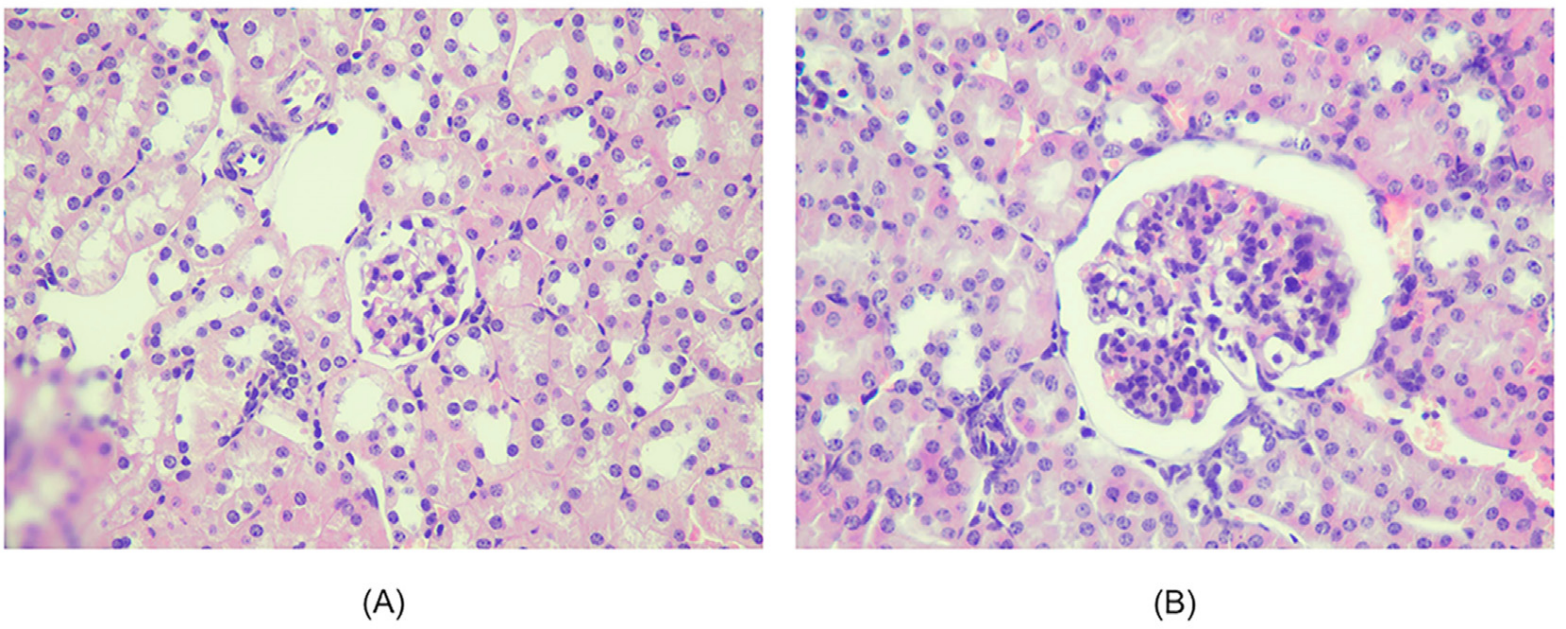
Histopathological view of renal sections in Healthy and treatment groups with HE (Hematoxylin and Eosin) staining, 400 HPF (A) The healthy group showed minimal mesangial matrix enhancement (B) The Placebo group showed hypercellular mesangial matrix, mesangial expansion, endocapillary hypercellularity dan infiltration of neutrophils.

The average expressions of IL-23 between the three groups were summarized in Table 2 and Figure 1. IL-23 at placebo group was higher compared to Healthy, Steroid, and *Nigella sativa* L. groups. The result of the IL-23 expression is presented as the average percentage of CD-11 b^+^ IL-23^+^. In Figure 2. The LSD multiple comparisons showed that the Placebo group has IL-23 level (5.17 ± 0.39) higher significantly than Steroid (3.20 ± 0.59%, p < 0.05) and *Nigella sativa* L. group (1.60 ± 0.57%, p < 0.001), and the IL-23 expression of *Nigella sativa* L. group is significantly lower than Steroid group (1.60 ± 0.57 vs 3.20 ± 0.5, p < 0.001).

### 3.5 Treg expression of lupus mice models

The mean absolute Treg cell count was higher in the Steroid group compare to *Nigella sativa* L. and Placebo groups. The results of the normality test of the data using the Shapiro-Wilk test showed that the data had a normal distribution (p > 0.05), while the results of the Levene homogeneity test showed that the data were not homogeneous (p < 0.05) so that the Brown-Forsythe substitute test was used for assessing at the differences between the two groups. From the results of the Brown-Forsythe test, a p-value of <0.001 (p < 0.05) was obtained, which indicated that there were at least two groups that were significantly different. Test between groups is also needed to determine the value of differences between groups. From the results of the Games-Howell multiple comparisons test, the mean was significantly higher in the Steroid group (314,182.38 ± 119,942.73) compared to the Placebo group (99,528.76 ± 56,395.11; p < 0.001) as well as with the *Nigella sativa* L. group (229,322.63 ± 54,432.00, p < 0.001). The mean absolute number of Treg cells in the *Nigella sativa* L. group was strongly higher when compared to the Placebo group as shown in Table 2.

### 3.6 Effect of *Nigella sativa* L. on histopathological renal tissue

**Figure 3:**
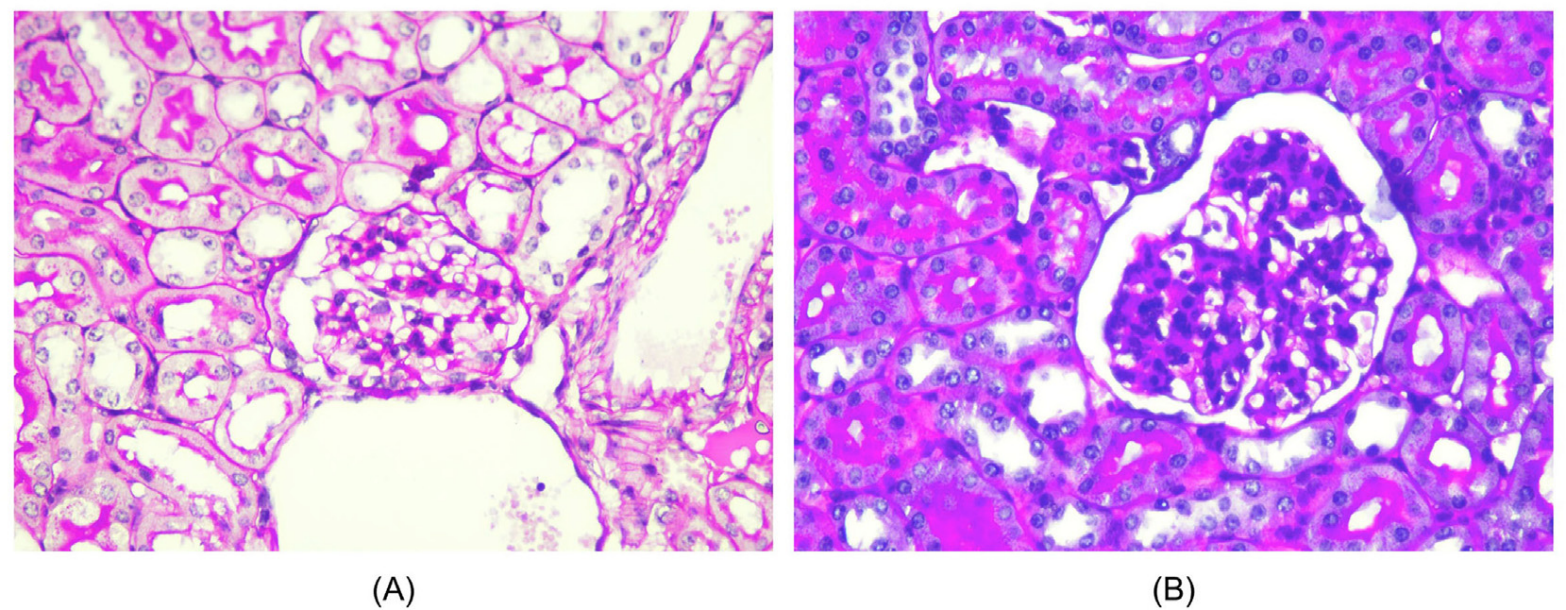
Histopathological slide of renal sections in Healthy and Placebo groups with PAS (Periodic acid–Schiff) staining, 400HPF. (A) The healthy group showed minimal mesangial matrix enhancement. (B) The placebo group showed hypercellular mesangial matrix, mesangial expansion, endocapillary hypercellularity dan infiltration of neutrophils.

**Figure 4:**
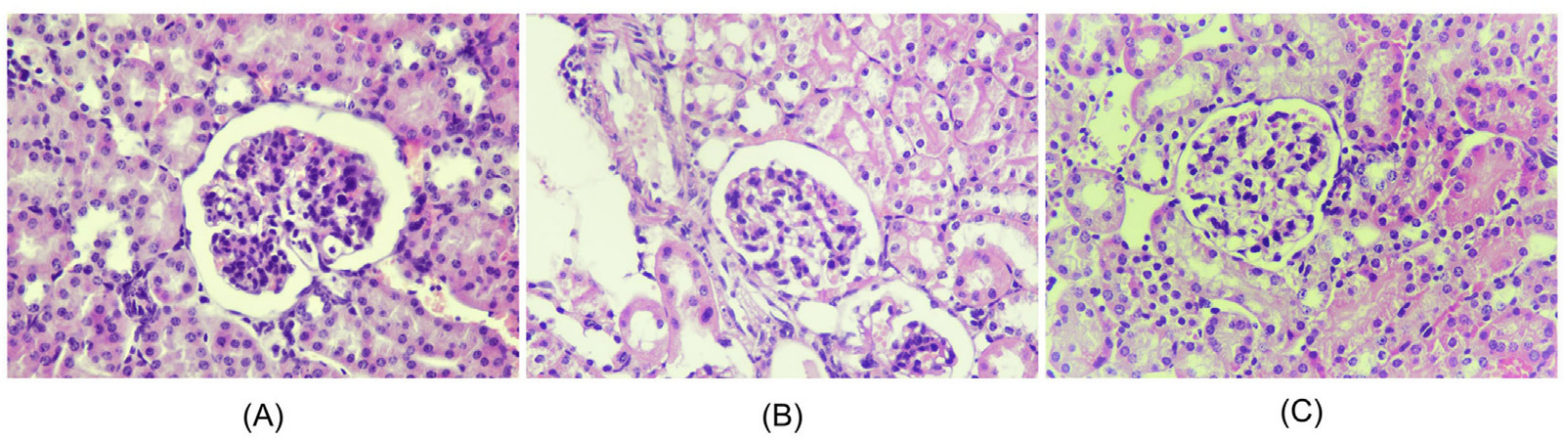
Histopathological slide of renal sections in Placebo, Steroid, and *N.sativa* groups (HE, 400 HPF). (A) The placebo group showed diffuse hypercellular mesangial, mesangial expansion, endocapillary hypercellularity dan infiltration of neutrophils. (B) The steroid group showed hypercellular mesangial matrix and mesangial expansion, without other abnormalities. (C) *Nigella sativa* L. group showed hypercellular mesangial matrix and minimally mesangial expansion appearance.

Histopathological changes of renal in all groups are shown in Figures 1, 2, 3, and 4. Microscopic examination of the Healthy group revealed normal renal glomeruli and minimal mesangial matrix enhancement (Figures 1 and 2). However, renal tissue damage is seen in the case of intraperitoneal injection of pristane. Diffuse hypercellular endocapillary, mesangial hypercellular, thickened capillaries and closed vascular endothelium were observed in the Placebo group (Figures 1 and 2). Mild histopathological lesions were observed in the Steroid and *Nigella sativa* L. groups (Figures 3 and 4). Observation of histological preparations showed that there was protective of renal structural damage in the Steroid and *Nigella sativa* L. groups. To assess the magnitude of the change in pathological scores, performed to assess the renal based on the ISN/RPS classification of LN by an anatomical pathologist (shown in Table 1). The Placebo groups showed a higher grade of LN (IV [IV–IV]) compared to the Healthy group (I [I–I], p < 0.05). The Steroid and *Nigella sativa* L. groups showed a lower grade of LN (III [II–IV] and II [II–III], p < 0.05) compared to the Placebo group. The severity of tissue damage in the renal of *Nigella sativa* L. group was considerably lower when compared to the Placebo and Steroid (p < 0.05) groups.

**Table 1:** The profile of subjects between mice lupus model and normal mice.

Subject's profile	Lupus model (N = 30)	Normal mice (N = 10)
Body weight (g) (post pristane)	28.11	38.16
Anti-dsDNA	55.15 ± 1.26	45.32 ± 2.56
Alopecia	30	0
Arthritis	30	0

The expression of anti-dsDNA IL-6, IL-23, IL-17, and Tregs on the lupus mice model is presented as the average value + standard deviation. All data were distributed normally (p > 0.05) and but not homogenous (p < 0.05).

### 3.7 Effect of *Nigella sativa* L. on Activity Indices

The effect of Placebo (carboxymethyl cellulose sodium 0.2%), Steroid (Prednisone), and *Nigella sativa* L. on renal Activity Index (AI) are shown in Table 2. Compared with the Healthy group, Placebo presented with significantly higher AI (4.5 [1–5] vs. 2 [0–3], p < 0.05). The Steroid group has lower AI when compared to the Placebo group (3 [2–4] vs. 4.5 [1–5], p < 0.05). *Nigella sativa* L. group significantly has lower AI when compared to Placebo or Steroid groups (2 [1–3] vs. 4.5 [1–5] or 3 [2–4], respectively, p < 0.05).

**Table 2:** The result of dsDNA, IL-17, IL-6, IL-23, and Treg expression on Healthy, Placebo, Steroid, and *Nigella sativa* L. group.

Groups	n	dsDNA		p-value
		$\bar{\text{x}}$**± SD**	Min-Max	
Healthy	10	45.32 ± 2.56^a^	41.36–47.88	<0.001∗
Placebo	10	57.88 ± 0.84^b^	56.12–58.87	
Steroid	10	52.86 ± 1.80^c^	50.99–55.91	
*Nigella sativa* L.	10	49.01 ± 1.17^d^	48.03–50.71	
		**IL-17**		
		$\bar{\text{x}}$**± SD**	**Min-Max**	
Healthy	10	0,89 ± 0,52^a^	0.19–1.52	<0.001∗
Placebo	10	2.51 ± 0.72^b^	1.73–3.73	
Steroid	10	1.39 ± 0.27^c^	0.85–1.76	
*Nigella sativa* L.	10	0.97 ± 0.31^d^	0.71–1.53	
		**IL-6**		
		$\bar{\text{x}}$**± SD**	**Min-Max**	
Healthy	10	0.21 ± 0.09^a^	0.09–0.34	<0.001∗
Placebo	10	2.39 ± 0.50^b^	2.03–3.59	
Steroid	10	0.49 ± 0.07^c^	0.38–0.59	
*Nigella sativa* L.	10	0.29 ± 0.06^d^	0.21–0.39	
		**IL-23**		
		$\bar{\text{x}}$**± SD**	**Min-Max**	
Healthy	10	1.59 ± 0.51^a^	0.73–2.33	<0.001∗
Placebo	10	5.17 ± 0.39^b^	4.68–5.87	
Steroid	10	3.20 ± 0.59^c^	2.36–4.35	
*Nigella sativa* L.	10	1.60 ± 0.57^d^	0.92–2.38	
		**Treg**		
		$\bar{\text{x}}$**± SD**	**Min-Max**	
Healthy	10	217.9 ± 52.0^a^	144.9–290.6	<0.001∗
Placebo	10	99,528.76 ± 56,395.11^b^	16,136.30–186,255.76	
Steroid	10	314,182.38 ± 119,942.73^c^	135,804.19–483,868.47	
*Nigella sativa* L.	10	229,322.63 ± 54,432.00^d^	149,168.74–326,112.20	

**∗**dsDNA: Kruskal-Wallis test, significant if α < 0.05; a,b,c,d Superscript showed the significant difference between the treatment group (Mann-Whitney U test); IL17:Kruskal-Wallis test, significant if α < 0.05; a,b,c,d Superscript showed the significant difference between the treatment group (Mann-Whitney U test); IL6:Kruskal-Wallis test, significant if α < 0.0; a,b,c,d Superscript showed the significant difference between the treatment group (Mann-Whitney U test); IL23:OnewayAnova, significant if α < 0.05; a,b,c Superscript showed the significant difference between treatment group of LSD multiple comparisons; Treg: Kruskal-Wallis test, significant if p = 0.05; a,b,c,d Superscript showed there is a significant difference among the treatment groups (multiple comparisons Games-Howell).

### 3.8 Effect of *Nigella sativa* L. on CD34+ cells

Means (±SD) of the data obtained in all groups are given in Table 3. The Placebo group showed significantly lower CD34+ cells compared to the Healthy group (46.0 ± 31.69 vs. 79.0 ± 9.94, p < 0.05). The steroid group has higher CD34+ cells significantly when compared to the Placebo group (57.0 ± 18.88 vs. 46.0 ± 31.69, p < 0.05). *Nigella sativa* L. group significantly has higher CD34+ cells when compared to Placebo and Steroid groups (76.5 ± 10.55 vs. 46.0 ± 31.69, and 57.0 ± 18.88, respectively, p < 0.05).

**Table 3:** Histopathological classification of LN, AI and CD34 cells in Healthy and Treatment groups.

Groups	n	Histopathological classification of LN (class)	p-value
		Median (min–max)	
Healthy	10	I (I–I)^a^	0.001∗
Placebo	10	IV (IV–IV)^d^	
Steroid	10	III(II–IV)^c^	
*Nigella sativa* L.	10	II (II–III)^b^	
		**AI**	
		**Median (min–max)**	
Healthy	10	2 (0–3)^a^	0.001∗
Placebo	10	4.5 (1–5)^d^	
Steroid	10	3 (2–4)^c^	
*Nigella sativa* L.	10	2 (1–3)^b^	
		**CD34**	
		Mean (min–max)	
Healthy	10	79.0 ± 9.94^a^	0.003∗
Placebo	10	46.0 ± 31.69^d^	
Steroid	10	57.0 ± 18.88^c^	
*Nigella sativa* L.	10	76.5 ± 10.55	

∗Kruskal-Wallis test; groups identified using different letters (a,b,c,d) are statistically significant (p < 0.05, Mann-Whitney test); LN: Lupus Nephritis; AI: Activity Indices; NIH: National Institutes of Health.

## 4 Discussion

*Nigella sativa* L. is well known as a cure for various diseases and had been proved to affects the immune system by modulating cytokines and Tregs (Prastiwi et al., 2015; Suprijono and Paramita, 2021; Shaterzadeh-Yazdi et al., 2018a, Shaterzadeh-Yazdi et al., 2018b; Arjumand et al., 2019). Cytokines such as IL-6, IL-23, IL-17, and Tregs that take a part in the SLE pathogenesis, *Nigella sativa* L. have been proven to modulate these cytokines and eliminate organ damage (including renal) in various diseases but the information about the effect on SLE is limited (Tackey et al., 2004; Dolff et al., 2011; Crispín et al., 2010; Rönnblom and Elkon, 2010). The present study is the first investigation that evaluates the effect of *Nigella sativa* L. as an immunomodulator and renal tissue preventive in SLE.

The IL-6 expression in *Nigella sativa* L. group is significantly lowest than other groups, which is similar to another animal study (Umar et al., 2012). Other in vivo study showed TQ in *Nigella sativa* L. able to inhibit TNF-α-induced IL-6 production (Amissah-Arthur and Gordon, 2010) and reduce the expression of IL-4, IL-5, IL-6, and TGF-b1 mRNA in the rat model of allergic airway inflammation (Shahzad et al., 2009). *Nigella sativa* L. group showed the lowest expression of IL-17 compared to other groups, which are consistent with study conducted in non-lupus diseases (Agustiarini et al., 2019). In contrast to the results of research on asthma patients conducted by Salem et al., which found that giving *Nigella sativa* L. could not significantly reduce IL-17 levels (Kheirouri et al., 2016; Salem et al., 2017). Others stated that *Nigella sativa* L. has no effect on Th17 which is the largest producer of IL 17A (Li et al., 2015).

This study showed that *Nigella sativa* L. group had the lowest expression of IL-23 compared to other treatment groups, which is supported by Tajmiri et al. (2016) study in Hashimoto's thyroiditis patients for 8 weeks. *Nigella sativa* L. decreased the expression of IL-23 and transformed growth factor-β (TGF-β) (Tajmiri et al., 2016). In this study, the absolute number of Treg in *Nigella sativa* L. group was higher than placebo. This study is in line with animal research on asthma and etherical Serovars Typhimurium (Barlianto et al., 2012; Ahmed et al., 2018). In contrast, Susanti et al. (2013) found no significant change in asthmatic rats treated with *Nigella sativa* L. (Susanti et al., 2013).

It is proposed that the reduction of the cytokine after the administration of TQ is due to its role to detain interleukin-1 receptor-associated kinase 1 (IRAK1) activation. The IRAK-1 receptor, a serine/threonine receptor, acts to regulate the Toll-Like Receptor (TLR) signal and transmits it to transcription factors for the synthesis of the pro-inflammation cytokines, Nuclear factor-kappa B (NF-k), and activator protein-1 (AP-1) pathways in macrophage, Dendritic cell (DC), and T lymphocyte cells. It also provokes the differentiation of T lymphocyte cells to the pro-inflammation immune response (M1 polarization), such as Th1 and Th17 by suppressing Treg activation. So inhibiting IRAK-1 activation via IRAK-1 receptor is the potential pathway for effective intervention in inflammation disease (Susanti et al., 2013; Maitra et al., 2009). TQ, as one of the bioactive compounds of *Nigella sativa* L., is capable to suppress or inhibit the activation of IRAK-1, so the DC activation is blocked and suppressing T cell lymphoid to proliferate to Th17 and down-regulate IL-17 expression. On the other hand, the suppression of IRAK-1 stimulates Treg expression by upregulating its synthesis (Hossen et al., 2017).

In the study of pediatric SLE patients showed that renal damage is one of the most common manifestations (Hiraki et al., 2008). The degree of renal damage was based on the ISN/RPS classification of LN. *Nigella sativa* L. groups showed a significantly lowest grade of renal damage compared to steroid and placebo groups. It shows that *Nigella sativa* L. can prevent renal damage better than steroid treatment. These findings agree with the findings of other investigators who observed supplementation of *Nigella sativa* L. on renal injury in Pristane induce arthritis in Rats (Faisal et al., 2015). We evaluate the renal score of NIH- Activity Indices (AI) to measure the progression of LN (Bajema, Wilhelmus, Alpers, Bruijn, Colvin, Cook, D'Agati, Ferrario, Haas and Jennette, 2018; Fava and Petri, 2019). *Nigella sativa* L. group showed significantly lower AI than the Placebo group. These results suggest that the administration of *Nigella sativa* L. may reduce the renal inflammation caused by autoimmune mechanisms. TQ reduced oxidative stress markers such as MDA and increased antioxidant content, including malondialdehyde (MDA); superoxide dismutase (SOD); glutathione (GSH); catalase (CAT); Glutathione-S-Transferase (GST), which have a critical role in renal damage (Shaterzadeh-Yazdi et al., 2018a, Shaterzadeh-Yazdi et al., 2018b).

The present study also assessed the potential effect of extract *Nigella sativa* L. by studying its effect on renal tissue damage, which was measured in terms of CD34+. Local CD34+ capillaries decrease in mouse models of renal disease associated with the severity of glomerular and tubulointerstitial lesions. *Nigella sativa* L. group shows significantly higher in CD34+ compared to Placebo group. This result showed that *Nigella sativa* L. can prevent the number of CD34+ glomerular capillaries decreased caused by the autoimmune process. *Nigella sativa* L. group is significantly increased CD34+ cells and resulted in lower AI than the Steroid and Placebo group. Several studies have shown an association between autologous transplantation of CD34-positive cells resulting in remission and reduced severity of symptoms in LN (Alchi et al., 2013; Su et al., 2013). Although this is the first study to evaluate the immunomodulatory and preventive effect on renal tissue damage of *Nigella sativa* L. in lupus, the absence of prior examination before treatment can be considered as a weakness of this study.

## 5 Conclusion

*Nigella sativa* L. can be consumed as the complementary medication for SLE, it has been proved to modulate the immune system by reducing the expression of proinflammatory response cytokine (IL-6, IL-17, IL-23) and increasing the expression of Tregs significantly, *Nigella sativa* L. also can prevent renal tissue damage in PIL model. Further studies in humans are needed regarding the effects of *Nigella sativa* L. as an immunomodulator and prevention of kidney disease, and evaluation of the negative effects, especially in humans.

## Declarations

### Author contribution statement

Zahrah Hikmah: Conceived and designed the experiments; Performed the experiments; Analyzed and interpreted the data; Contributed reagents, materials, analysis tools or data; Wrote the paper.

Anang Endaryanto: Conceived and designed the experiments; Performed the experiments; Wrote the paper.

I Dewa Gede Ugrasena: Conceived and designed the experiments; Contributed reagents, materials, analysis tools or data; Wrote the paper.

Anny Setijo Rahaju: Analyzed and interpreted the data; Wrote the paper.

Syaiful Arifin: Contributed reagents, materials, analysis tools or data; Wrote the paper.

### Funding statement

This research did not receive any specific grant from funding agencies in the public, commercial, or not-for-profit sectors.

### Data availability statement

Data will be made available on request.

### Declaration of interests statement

The authors declare no conflict of interest.

### Additional information

No additional information is available for this paper.
